# Integrin α_v_ contributes to the regulation of vascular smooth muscle cell stiffness

**DOI:** 10.1038/s41598-026-38948-z

**Published:** 2026-02-07

**Authors:** Rümeyza Bascetin, Ekaterina Belozertseva, Véronique Regnault, Alexandre Raoul, Xiao Liu, Caterina Maria Tone, Ali-Akbar Karkhaneh-Yousefi, Huguette Louis, Zhor Ramdane-Cherif, Cindy Lerognon, Stéphane Avril, Adam Lacy-Hulbert, Daniel Henrion, Emmanuelle Lacaze, Pascal Challande, Zhenlin Li, Patrick Lacolley

**Affiliations:** 1 Université de Lorraine, INSERM, DCAC, Nancy, F-54000 France; 2 Sorbonne Université, CNRS, Institut des Nano-Sciences de Paris (INSP), Paris, 75005 France; 3 Physics Department, University of Calabria, ponte P.Bucci, Rende (CS), Rende, Cosenza (CS) Italy; 4 Mines Saint-Etienne, Université Jean Monnet, INSERM, Sainbiose, Saint- Etienne, 1059 France; 5 Department of Immunology, University of Washington, Seattle, WA 98109 USA; 6 University of Angers, INSERM U1083, CNRS UMR 6015, MITOVASC, SFR ICAT, Angers, 49000 France; 7 Sorbonne Université, CNRS, Institut Jean Le Rond d’Alembert, 4 place Jussieu, Paris, 75005 France; 8 Sorbonne Université, CNRS, INSERM, IBPS, Biological Adaptation and Ageing, Paris, France; 9 Faculté de Médecine, Inserm U1116, 9 avenue de la forêt de Haye, Vandoeuvre-lès-Nancy Cedex, 50184, 54505 CS France

**Keywords:** Vascular smooth muscle cells, Integrins, Cell stiffness, Focal adhesion, Atomic force microscopy, Finite element method, Cell biology, Diseases, Physiology

## Abstract

Arterial stiffening is influenced by the organization of focal adhesions in vascular smooth muscle cells (VSMCs). We investigated the contribution of α_v_ integrins to both arterial wall stiffness (Young’s modulus measured by echography) and VSMC stiffness (assessed by atomic force microscopy). Mice with VSMC-specific deletion of α_v_ integrins (α_v_^SMKO^) were compared with controls at baseline and following angiotensin II infusion. Unstimulated cultured α_v_-deficient (α_v_-KD) VSMCs exhibited higher stiffness than controls, with a further increase after angiotensin II. To interpret AFM measurements performed at shallow indentation depths, we developed a computational model of VSMC nanoindentation. Simulations showed that higher apparent Young’s moduli at shallow indentation fall within the experimental range of α_v_-KD cells. These cells also displayed enhanced actin polymerization, further amplified by angiotensin II through the formation of cortical F-actin. In vivo, arterial pressure and wall elastic modulus were similar between α_v_^SMKO^ and control mice at baseline and after angiotensin II, despite α_v_^SMKO^ mice exhibiting lower elastin and higher collagen content under angiotensin II. Together, these findings indicate that the comparable increase in arterial stiffness observed in α_v_^SMKO^ mice under angiotensin II is driven primarily by elevated VSMC stiffness resulting from cortical actin redistribution, which outweighs extracellular matrix changes.

## Introduction

Integrins are non-covalently associated heterodimeric cell-surface adhesion receptors that mediate vital bidirectional signals during morphogenesis, tissue remodeling and repair. It is well established that α_v_ integrins are widely expressed in many tissues throughout development and mediate the process of angiogenesis which is central to immune regulation and inflammation by vascular cells^1^. Conditional inactivation of the α_v_-subunit in endothelial cells of mice showed that integrin α_v_ is important for heart- and large vessels remodeling during development^2^. The integrin α_v_ subunit is highly expressed in vascular smooth muscle cells (VSMCs) and implicated in the control of contractility, cell adhesion, proliferation/migration and apoptosis^3^. Conditional inactivation of the α_v_ subunit in VSMCs decreases angiotensin II (Ang II)-induced vascular fibrosis via an α_v_ integrin/transforming growth factor beta (TGF-β)/CD109 axis^4^. However, whether α_v_-containing integrins on VSMCs have the potential to modulate VSMC stiffness with consequences on arterial stiffening remains to be elucidated. Arterial stiffness is an independent predictive indicator for total and cardiovascular mortality, especially in subjects with hypertension, heart failure and other age-associated diseases^5^. Arterial stiffening and distensibility of the vascular wall are dependent on the relative contribution of its two most prominent scaffolding proteins of the extracellular matrix (ECM): collagen and elastin. The relative content of these molecules is normally held stable by a slow, but dynamic, process of production and degradation. Dysregulation of this balance, mostly because of inflammation, leads to an overproduction of abnormal collagen and a decrease of normal elastin, which contribute to vascular stiffness^5^. Increased luminal pressure, or hypertension, also stimulates activation of TGF-β leading to excessive collagen production^3^. TGF-β1 and Ang II have been shown to be critical for cardiac remodeling and tissue repair^6,7^. Ang II induces fibronectin, laminin, and TGF-β1 mRNA transcription via its Ang II type 1 receptor (AT1) in cardiac fibroblasts and α_v_ integrins in VSMCs^4,8,9^.

In response to changes in their environment, VSMCs switch from their quiescent contractile “mature” phenotype to “embryonic” dedifferentiated phenotype^10^. This phenotypic modulation of VSMCs plays a pivotal role in arterial stiffening associated with aging^11^. VSMC–ECM interactions are key molecular/cellular mechanisms in mechanotransduction. VSMC stiffness appears to be crucial role in the regulation of arterial stiffening through its role in cytoskeletal architecture and focal contacts to the ECM^12,13^.

The role of the α_v_ integrin subunit in arterial mechanical properties remains insufficiently defined. To investigate its contribution to arterial stiffness, including extracellular matrix remodeling, focal adhesion dynamics, and VSMC intrinsic stiffness, we used a mouse model with conditional deletion of α_v_ in VSMCs (α_v_^SMKO^). We hypothesized that α_v_ integrins regulate vascular stiffness not only through ECM alterations but also by modulating VSMC mechanical properties. Our aim was to assess the role of α_v_ integrins in adult mice and in primary aortic VSMCs under basal conditions and following Ang II stimulation.

## Results

### VSMC stiffness

**Fig. 1:**
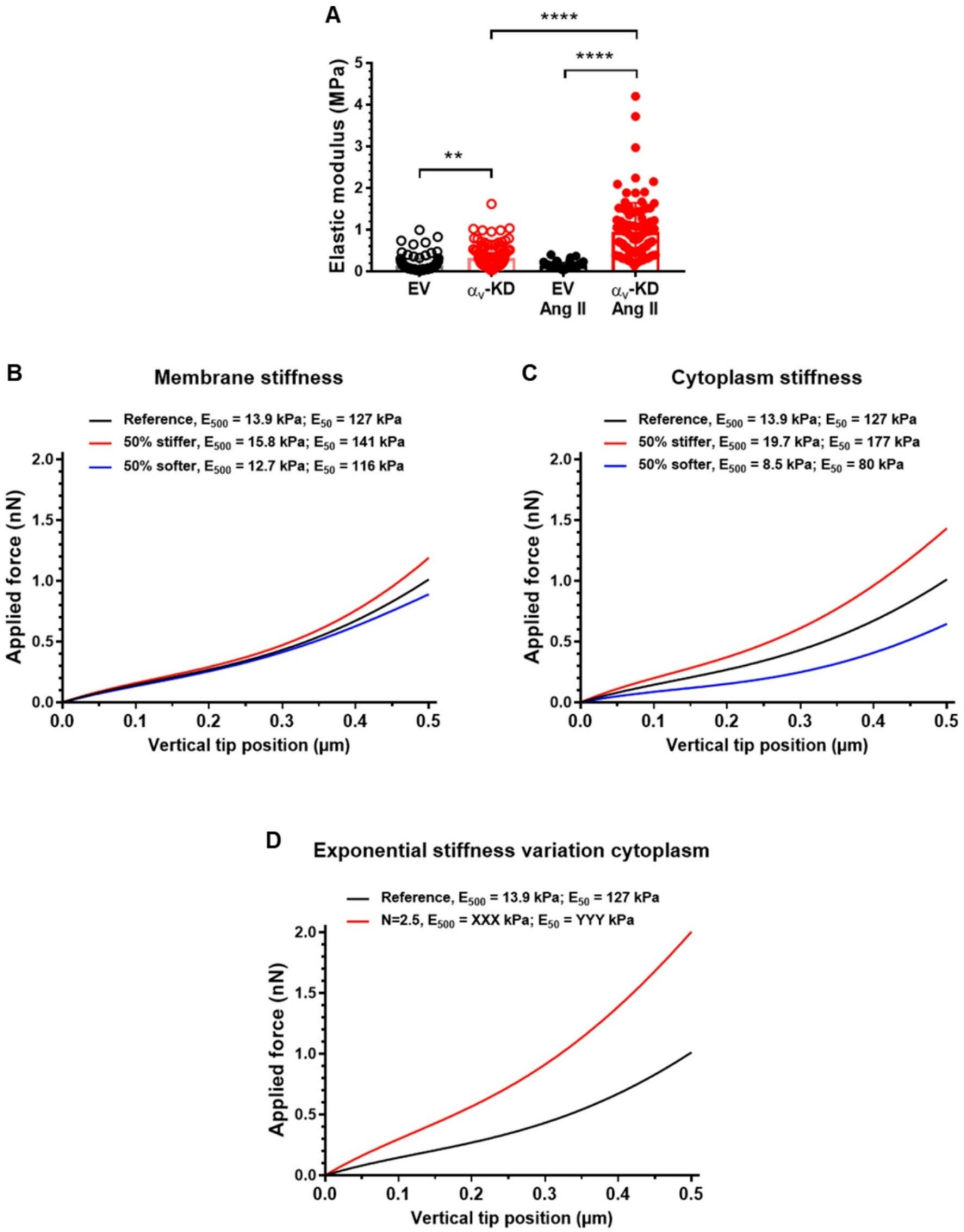
Cell stiffness of VSMCs from Ctrl and α_v_^SMKO^ mice. (A) Elastic modulus values from non-treated EV (*n* = 122 values), non-treated α_v_-KD (*n* = 120 values), Ang II-treated EV (*n* = 64 values) and Ang II-treated α_v_-KD (*n* = 99 values) mouse VSMCs. (B) Simulated force-displacements curves for different values of cell membrane stiffness. (C) Simulated force-displacements curves for different values of cytoplasm stiffness. (D) Simulated force-displacements curves for an exponential variation in cytoplasmic stiffness.

We experimentally quantified VSMC stiffness using atomic force microscopy (AFM) nanoindentation, following a protocol comparable to that employed in previous studies assessing rodent VSMC mechanics^12–15^. For technical reasons related to cell adhesion and tip stability, the maximal indentation depth was limited to approximately 50 nm. The values of indentation Young’s modulus obtained within this indentation range are displayed on Fig. 1A. We observed that at baseline the indentation Young’s modulus was significantly higher in α_v_-deficient (α_v_-KD) cells compared with empty vector (EV) cells with an increase of a factor more than 2 (0.32 ± 0.03 MPa vs. 0.14 ± 0.02 MPa). While 48 h of Ang II treatment did not induce significant change in EV cells (0.13 ± 0.01 MPa vs. 0.14 ± 0.02 MPa), it significantly increased the indentation Young’s modulus in α_v_-KD cells by a factor about 3 (0.95 ± 0.07 MPa vs. 0.32 ± 0.03 MPa).

### Computational study of VSMC stiffness in a nanoindentation test

The 50 nm indentation performed in our study is shallower than the ~ 500 nm indentation commonly used to probe deeper cytoplasmic mechanics in the literature. Conducting additional AFM experiments at greater indentation depths was not feasible. Therefore, we developed a computational finite-element (FE) model to evaluate how indentation depth influences the apparent stiffness of VSMCs and to verify whether the biological conclusions remain consistent across indentation regimes. Force displacement curves resulting from the FE simulation of AFM indentation on VSMCs are displayed in Fig. 1B, C and D. Each of these figures show the response for the reference cell have a cytoplasmic elastic modulus of 600 Pa and a membrane elastic modulus of 3600 kPa.

For each reference curve, we identified the indentation Young’s modulus by linear regression of the Sneddon’s model as for the experimental study. We report the obtained value onto the figure first by using only the force displacement data with tip displacements in the range [0–50 nm] and then with tip displacements in the range [0–500 nm]. The former apparent Young’s modulus is denoted E_50_ and the latter is denoted E_500_. It can be noticed that E_50_ is usually 10 times larger than E_500_, showing that the indentation apparent Young’s modulus of a cell depends strongly on the depth of penetration. Significantly larger values are obtained if we have only a shallow indentation. This may be explained by the larger influence of the membrane on the response for shallow indentation, the membrane being much stiffer than the cytoplasm.

We performed a sensitivity analysis using FE simulations to investigate the effects of cytoplasmic and cell membrane stiffness on the force-displacement response during AFM indentation. Figure 1B and C show the resulting force-displacement curves for the varying cytoplasmic and membrane stiffness. It can be observed that the indentation curves vary more significantly when varying the stiffness of the cytoplasm than the stiffness of the cell membrane. This means that the cytoplasm stiffness has more influence on the indentation response than the membrane stiffness, which can be explained by the depth of penetration of 500 nm.

Additionally, we conducted another simulation to explore a hypothesis for the increased stiffness observed in α_v_-KD VSMCs. It is known that α_v_ integrins promote actin polymerization within the cytoplasm and lead to actin accumulation near the cell cortex. This cortical actin accumulation is expected to increase local material stiffness near the cell membrane. To consider this remodeling effect, we introduced a spatially varying elastic modulus in the cytoplasm, assigning a higher stiffness near the membrane. Specifically, we increased exponentially the elastic modulus from its reference value of 600 Pa at the center to 1500 Pa at the cell membrane. Figure 1D shows the force-displacement curve obtained after the FE simulation. The higher apparent indentation Young’s modulus in the spatially variable stiffness fall within the range of experimentally observed Young’s moduli for α_v_-KD VSMCs. These findings suggest that localized cortical stiffening due to actin accumulation could contribute to the increased apparent Young’s modulus of α_v_-KD VSMCs.

### Integrin-mediated signaling pathway

**Fig. 2:**
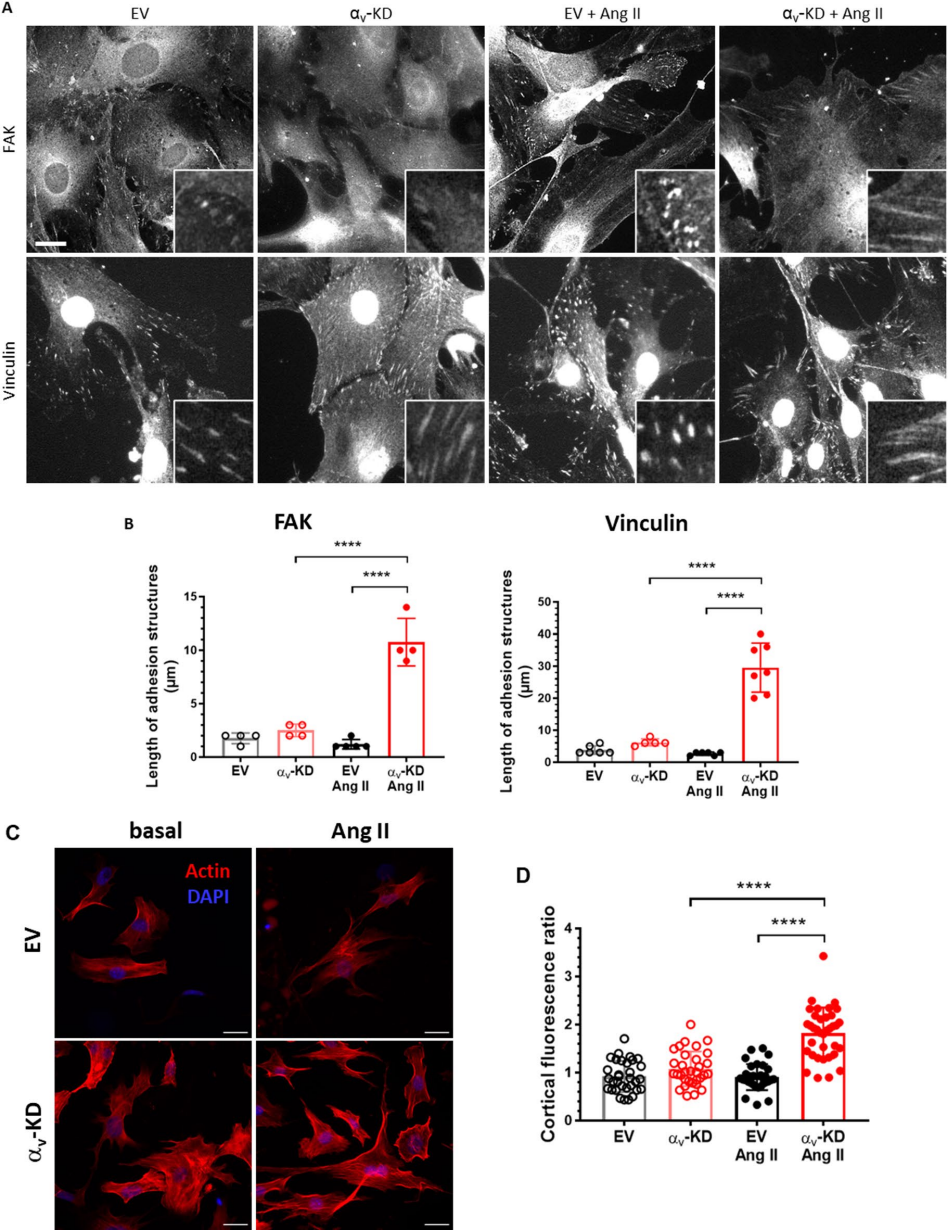
Focal adhesion proteins and actin organization in VSMCs from Ctrl and α_v_^SMKO^ mice. (A) Representative images of immunostained FAK and vinculin. Scale bar: 20 μm. Insets are magnification of adhesion sites. (B) Measurement of lengths of adhesion structures in magnified images. (C) Actin filament in murine primary VSMCs. Representative images of actin filaments stained with phalloidin (red) and DNA stained with DAPI (blue). Scale bar: 50 μm. (D) Cortical fluorescence ratio: ratio of red fluorescence intensity near wall on mean intensity along lines.

Actin organization upon matrix rigidity sensing is dependent of adhesion structures. We thus examined organization of adhesion structures by immunostaining focal adhesion kinase (FAK) and vinculin (Fig. 2A). We observed small adhesion structures mostly at the edge of the cells (length around 2 μm for FAK and 4 μm for vinculin) for the control condition without Ang II (Fig. 2B). For α_v_-KD VSMCs, cells seemed to form longer adhesion structures (length around 2.5 μm for FAK and 6 μm for vinculin) although differences are not significant. After treatment with Ang II, control cells exhibited similar small adhesion structures (length around 2 μm). The length of the adhesion structures at the edge of αv-KD cells treated with Ang II is even higher, at around 30 μm.

Actin polymerization had a major influence on the cell stiffness^16,17^. We examined the level of filamentous actin by phalloidin staining in VSMCs. EV cells, with or without Ang II stimulation, presented a diffuse staining of actin filaments within the cytoplasm. α_v_-KD VSMCs presented well defined stress fiber in the cytoplasm and were highly stained with phalloidin compared to EV cells (Fig. 2C). After Ang II stimulation, α_v_-KD VSMCs organized more peripheral cortical actin. The cortical ratio was significantly higher in α_v_-KD VSMCs with Ang II (1.82 ± 0.09) than in the others groups (EV: 0.91 ± 0.06, EV with Ang II: 0.91 ± 0.05, α_v_-KD: 1.08 ± 0.06) (Fig. 2D). Taken together, the responsibility and adaptability of α_v_-KD cells to environment and Ang II treatment were lower compared to control cells.

### Histological and morphological changes

Ang II produced a significant increase in media cross sectional area (MCSA) in α_v_^SMKO^ and control (Ctrl) mice (Table 1). Elastin density was smaller in α_v_^SMKO^ compared with Ctrl mice at baseline. Collagen density did not differ between the two groups of mice at baseline. Ang II produced a decrease in elastin in Ctrl mice. Collagen was increased in Ang II-treated α_v_^SMKO^ and Ctrl mice. Collagen was significantly lower in Ang II-treated α_v_^SMKO^ mice compared with Ang II-treated Ctrl mice.

**Table 1:** Mechanical properties and composition of the carotid artery.

-	Ctrl	α_v_^SMKO^	Ctrl Ang II	α_v_^SMKO^ Ang II
*Conscious mice*				
Number Weight (g) SAP (mmHg)	8 28 ± 2 109 ± 2	12 29 ± 1 101 ± 2	8 26 ± 1 130 ± 5*	8 31 ± 1 126 ± 5*
*Anaesthetized mice*				
Number Weight (g) SAP (mmHg)	18 28 ± 1 102 ± 2	12 29 ± 1 93 ± 2	8 26 ± 1 118 ± 5*	8 31 ± 1 112 ± 5*
DAP (mmHg)	70 ± 2	59 ± 2^§^	77 ± 4	80 ± 3*
MAP (mmHg)	81 ± 2	71 ± 2^§^	91 ± 4	90 ± 3*
PP (mmHg)	32 ± 3	34 ± 2	42 ± 2*	33 ± 3
HR (beats/min)	393 ± 14	410 ± 9	498 ± 12*	486 ± 17*
*Parameters at MAP*				
Dia (µm)	512 ± 13	519 ± 19	619 ± 35*	576 ± 19
Dist (10^− 3^ mmHg^− 1^)	10.3 ± 0.6	13.3 ± 0.7^§^	4.3 ± 0.4*	4.4 ± 0.4*
Einc (kPa) WS (kPa)	490 ± 49 231 ± 17	363 ± 38 200 ± 18	1007 ± 161* 227 ± 38	1011 ± 149* 223 ± 21
*Parameters in common range*				
Dia_80−100_ (µm)	533 ± 14	567 ± 12	619 ± 39*	575 ± 19
Dist_80−100_ (10^− 3^ mmHg^− 1^)	8.2 ± 0.4	6.7 ± 0.3^§^	4.5 ± 0.2*	4.4 ± 0.3*
WS_800−1000_ (kPa)	327 ± 13	305 ± 13	216 ± 19*	206 ± 11*
*Histology of the CA*				
MCSA (mm^2^ 10^− 3^)	20 ± 1	21 ± 1	35 ± 3*	29 ± 2*
Elastin density (%)	50 ± 1	44 ± 1^§^	37 ± 2*	41 ± 1
Collagen density (%)	25 ± 1	26 ± 1	36 ± 1*	30 ± 1*^#^

Values are means ± SEM. CA, carotid artery; Ctrl, control mice; DAP, diastolic arterial pressure; MAP, mean arterial pressure; PP, pulse pressure; Dia, diameter; Dist, distensibility; Einc, incremental elastic modulus at MAP; WS, wall stress; Dia_80−100_ mean diameter in 80–100 mmHg range; Dist_80−100_ mean distensibility in 80–100 mmHg range; WS_800−1000_ mean wall stress in 800–1000 kPa Einc range; MCSA, media cross sectional area. *P < 0.05 versus control of the same strain. ^§^*P* < 0.05 versus Ctrl. ^#^*P* < 0.05 versus treated Ctrl.

### In vivo arterial mechanical parameters

Under basal conditions, systolic arterial pressure (SAP) was not different between α_v_^SMKO^ and Ctrl mice (Table 1) in both conscious and anaesthetized animals. Diastolic arterial pressure (DAP) and mean arterial pressure (MAP) were lower in anaesthetized α_v_^SMKO^ compared to Ctrl mice. There was no difference in pulse pressure (PP) and HR. Ang II produced a significant increase in SAP and heart rate (HR) in α_v_^SMKO^ and Ctrl mice. DAP and MAP were also increased in Ang II-treated α_v_^SMKO^ mice compared with untreated α_v_^SMKO^ mice while PP was increased in Ang II-treated Ctrl mice compared with untreated Ctrl mice.

Carotid artery luminal diameter (Dia), incremental elastic modulus (Einc) and circumferential wall stress (WS) at MAP were similar at baseline between α_v_^SMKO^ and Ctrl mice. Ang II produced an increase in Dia in Ang II-treated Ctrl mice (Table 1). Distensibility (Dist) was lower and Einc was higher in Ang II-treated α_v_^SMKO^ and Ctrl mice compared with untreated mice without changes in mean WS. Dist at MAP was increased in α_v_^SMKO^ mice compared with Ctrl mice at baseline.

**Fig. 3:**
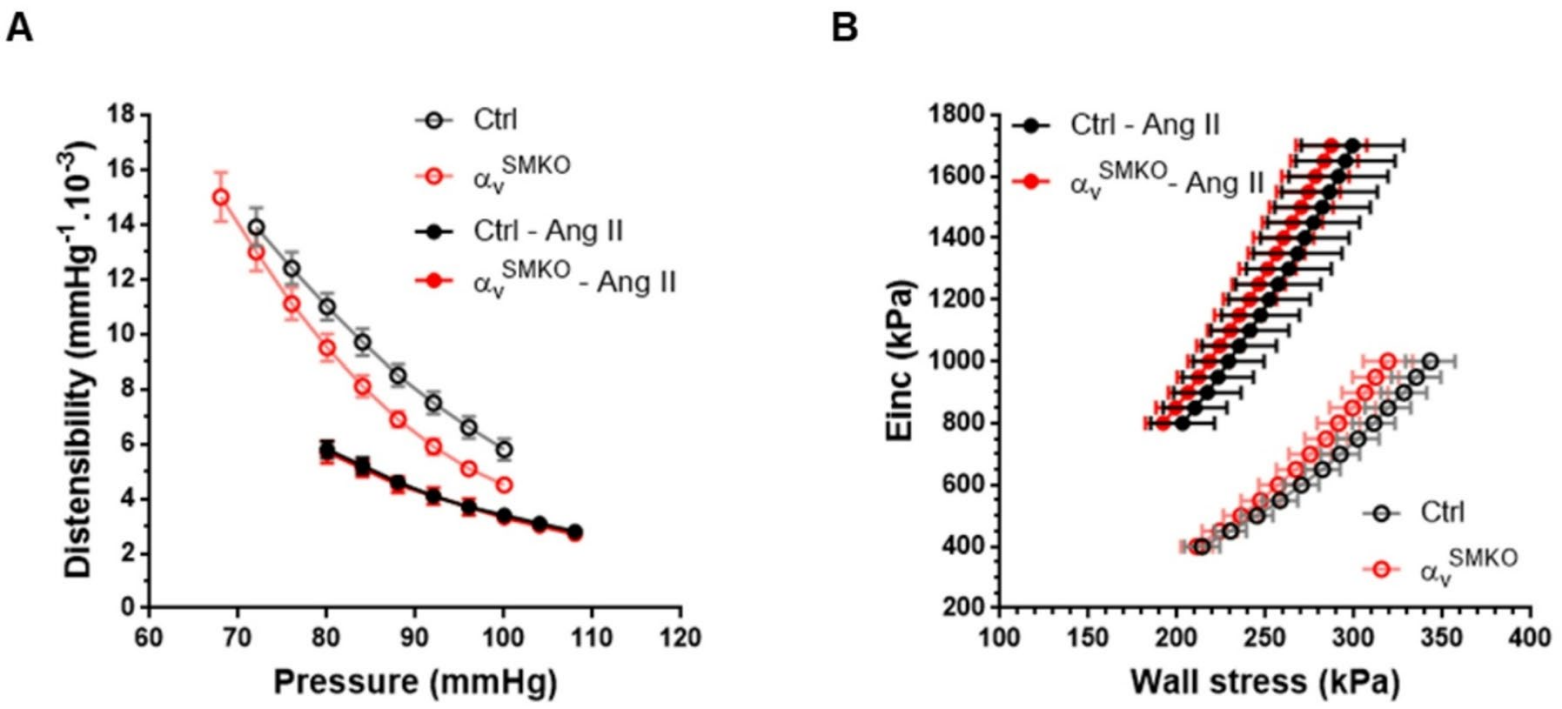
Arterial stiffness of the carotid artery in Ctrl and α_v_^SMKO^ mice. Arterial pressure -distensibility curves (A) and wall stress-Einc curves (B) in non-treated Ctrl (*n* = 18), non-treated α_v_^SMKO^ (*n* = 12), Ang II-treated Ctrl (*n* = 8) and Ang II-treated α_v_^SMKO^ (*n* = 8) mice.

Dist/arterial pressure (AP) curves are shown in Fig. 3A and Einc/circumferential WS curves are shown in Fig. 3B. At baseline, the Dist-AP curve of α_v_^SMKO^ mice was under that of Ctrl mice. The shift was assessed by a lower mean Dist in the common range (Dist_80−100_) (Table 1). The Einc/WS curves did not differ between the two groups of mice at baseline. The Dist-AP curves were significantly shifted downwards in response to Ang II in both groups. The Einc-WS curves of Ang II-treated α_v_^SMKO^ and Ctrl mice were significantly shifted leftwards, as assessed by the mean WS in the common range (WS_800−1000_) (Table 1). Dist-AP and Einc-WS curves did not differ between the two groups after Ang II treatment.

## Discussion

This study demonstrates that α_v_-KD VSMCs exhibit increased cell stiffness at baseline and in response to Ang II. FE simulations indicate that similar stiffness increase could result from localized cortical actin accumulation. The main functional consequence is that αv integrins help regulate arterial stiffness, acting alongside ECM remodeling and focal adhesion activation in response to elevated pressure.

We used the SM22-CreER^T2^(ki) strain to inactivate loxP-flanked α_v_ gene with up to 60% decrease of α_v_ mRNA level in vessel^4^. Administration of Ang II is a well-established model of arterial stiffening by stimulating profibrotic and proinflammatory gene expression^18^. We previously reported that Ang II or cyclic stretch induced a marked upregulation of α_v_ subunit gene^19,20^. Ang II was administered at 1000 ng×kg^− 1^×min^− 1^ for 28 days, based on established protocols reliably inducing arterial stiffness in the murine aorta^18,21,22^. A moderate elevation of blood pressure (by less than 30 mmHg) clarifies the contribution of cellular stiffness, particularly that of α_v_ integrin, to the overall stiffness of the arterial wall. In cell experiments, a 48-h treatment window has been used since it is long enough to see biological changes at the protein level but short enough to avoid confounding variables such as over-confluency or nutrient depletion.

Stiffening of the aorta and other central arteries is a major risk factor for cardiovascular morbidity and mortality, as well as for aortic dissection^23,24^. Our different previous works indicate that arterial wall stiffness has multi-factorial dependence on parameters such as intermediate filament proteins, intercellular and focal adhesion markers and membrane receptors (integrins) and highly depends on VSMC internal cytoskeleton organization and their interactions with ECM^19,25–27^. Until now, the effect of α_v_ integrin has only been reported regarding fibrosis in several tissues^28–30^. Our previous works have demonstrated that conditional inactivation of the α_v_ subunit in VSMCs decreased Ang II-induced vascular fibrosis via CD109 downregulation of TGF-β signaling and have highlighted decreased collagen content in the media of carotid arteries of α_v_^SMKO^ mice compared with Ctrl mice^4^. This effect appears independent of hemodynamic changes since AP and WS are similar in both strains treated with Ang II.

In the last decade, several authors reported that increased aortic stiffness in aging or hypertension is attributable not only to changes in ECM but also to intrinsic stiffness of VSMCs^12,13^. VSMC stiffness depends on dynamic cytoskeletal polymerization and depolymerization and increased adhesion forces^12^. Single-cell measurements using AFM is a common means to analyze cell topography and stiffness (local apparent Young’s modulus)^31^. We used a nanoindentation protocol similar to other published studies where VSMC stiffness of rodents was also measured with AFM^12–15^. The obtained indentation Young’s modulus VSMC (Einc) is representative of the cell stiffness. At baseline, VSMC Einc is higher in α_v_-KD cells compared to EV cells (2.3-fold increase). After Ang II treatment, VSMC Einc increases only in α_v_-KD cells (3-fold) and is 7-fold higher than in EV cells. The absence of increased stiffness in EV cells is unlikely to stem from Ang II-induced tachyphylaxis. Our previous findings of sustained molecular changes observed at 48 h^4^ indicate that VSMCs remain responsive to the stimulus, undergoing active long-term structural remodeling rather than receptor desensitization. This indicates that α_v_ is useful to maintain a low level of VSMC stiffness in response to Ang II. These results are consistent with Sehgel & al who showed that VSMCs from hypertensive rat present higher cell stiffness when stimulated with Ang II^13^.

Because technical constraints limited AFM indentation to 50 nm, which is shallower than the ~ 500 nm typically used, we developed a FE model to assess how indentation depth affects apparent VSMC stiffness and to confirm the consistency of our biological conclusions. Our FE simulations showed that using an indentation of 50 nm leads to much higher values (E_50_ of about 100 kPa), compared to larger indentations (E_500_ of about 10 kPa for an indentation of 500 nm). For every simulation, the ratio E_50_ to E_500_ was about 9. This result confirmed that it is not possible to compare values from different publications if the protocols are not strictly identical. In addition, thanks to the FE simulations, we were able to quantify the influence of various mechanical parameters of the cell. Increase in cytoplasm stiffness or membrane stiffness cannot account for the increase in Einc of α_v_-KD cells following Ang II. It is known that α_v_ integrins promote actin polymerization within the cytoplasm and lead to actin enrichment near the cell cortex. This cortical actin accumulation is expected to increase local stiffness near the cell membrane. To simulate this remodeling effect numerically, we introduced a spatially varying shear modulus in the cytoplasm of the FE model, assigning a higher stiffness near the membrane. Specifically, the shear modulus increased exponentially from its reference value close to the substrate to 2.5 times that value at the cell membrane proximity. Thus, we examined the role of F-actin distribution as our data showed that α_v_-KD VSMCs exhibit markedly increased stress-fiber formation, which is further enhanced by Ang II through the development of cortical actin. This distribution is different in the Ang II-treated α_v_-KD cells compared to the others: there is more cortical F-actin which is a thin layer of actin gel attached to the plasma membrane and important for cell shape, adhesion and migration among other functions^32^. This is confirmed by the calculation of the cortical fluorescence ratio which is higher in α_v_-KD cells compared to EV cells, which are compatible with higher stiffness near the membrane. These results are consistent with previous reports indicating that actin content and F/G actin ratio are related to VSMC stiffening and/or to aortic stiffening^5,12,15,25,33–35^. This relation to aortic stiffening links VSMC properties to ECM composition, as higher collagen content and lower elastin content are the main contributors of increased matrix stiffness. These findings collectively highlight the dynamic interplay between cortical actin organization, cell stiffness, and the mechanical properties of the surrounding matrix, consistent with previous reports^16,36,37^.

Several studies showed that stiffer substrates lead to larger focal adhesions, which are associated with higher cellular tension and the presence of thick actin stress fibers^38,39^. This is balanced by the maturation of focal adhesions. In line with these results, knocking down αv integrins in VSMCs resulted in significantly longer adhesion structures, amplified by a factor 4, as assessed by FAK and vinculin, in response to Ang II treatment. These modifications are consistent with a transition from stable focal adhesion to fibrillar adhesion. This is in line with the increase in cortical F-actin observed following Ang II treatment. Our data demonstrate the significant role of αv integrins in focal adhesion dynamics, as we have previously shown that knocking down αv integrins does not lead to an increase in α_5_β_1_ integrin.

ECM fibers are known to play a major role on arterial stiffness. In basal conditions, there is less elastin in α_v_^SMKO^ mice compared to Ctrl mice. The Ang II treatment induces more ECM changes in favor of stiffening in Ctrl mice compared to α_v_^SMKO^ mice (decrease in elastin content, higher increase in collagen content). Thus, the increase in arterial stiffness after Ang II infusion observed in Ctrl mice is not explained by changes in α_v−_mediated focal adhesion activation or elasticity at the cell level but rather by changes in ECM: increase in collagen density and decrease in elastin^40^.

Dist is an indicator of the mechanical behavior of the vessel including wall properties and geometry. Under basal conditions, in a common range of AP, Dist is lower in α_v_^SMKO^ mice compared with Ctrl mice while Dist at MAP is higher (Table 1). This latter result is explained by the fact that MAP is significantly lower in α_v_^SMKO^ mice. The lower Dist in the common range of AP can be explained by a lower elastin content. Einc/WS curves give the ability to compare the arterial wall stiffness of several groups. In the present study, there is no difference between α_v_^SMKO^ and Ctrl mice, both in basal conditions and after Ang II treatment. Ang II produces a similar increase of arterial wall stiffness assessed by the leftward shift of curves. This indicates that the depletion of α_v_ does not lead to a significant modification of arterial wall stiffness, neither before, nor after Ang II stimulation.

To recapitulate, the equivalent arterial wall stiffness based on Einc/WS curves in both mouse strains at baseline and the similar stiffness increase following Ang II treatment are explained by the three components: ECM changes, focal adhesion activation and cell stiffness. Interestingly, changes in these 3 determinants differ in the two mouse strains. At baseline, the higher VMSC stiffness in α_v_^SMKO^ mice compared to Ctrl mice appear to be compensated by a lower elastin content. The Ang II–induced increase in arterial stiffness in control mice is driven by reduced elastin and increased collagen within the ECM, without alterations in focal adhesion activation or cell stiffness. In contrast, the comparable stiffening observed in α_v_^SMKO^ mice under Ang II treatment results primarily from increased VSMC stiffness, linked to fibrillar adhesion formation and cortical actin redistribution, which outweighs the matrix changes seen in Ctrl mice.

## Conclusion

Our findings demonstrate that loss of αv integrins in VSMCs under chronic Ang II exposure drives a pronounced stiff VSMC phenotype, as revealed by AFM. This stiffening arises from dynamic remodeling of focal adhesions and cytoskeletal architecture, independent of extracellular matrix changes. By integrating a computational nanoindentation model, we further show that shallow indentations markedly amplify apparent stiffness and that cortical actin redistribution provides a mechanistic basis for this effect.

The cortical actin network is a dynamic and responsive determinant of the VSMC stiff phenotype that could be modulated through several distinct drugs targeting specifically the sub-membranous actin network such as depolymerizing agents, decoy peptides, or inhibitors of PAI-1, AMPK, and Src^41–46^. Antagonists of the renin angiotensin alone or in association remain the gold standard therapies of essential hypertension and heart failure. The observed resistance of these treatments to arterial stiffness, particularly in ageing, could be related to non-pressure-dependent effects on cell stiffness, particularly on several common signaling pathways linking α_v_ integrins to the actin network. Together, these results identify cortical actin reinforcement as a previously unrecognized contributor to VSMC stiffening, with potential relevance for isolated systolic hypertension and age-related arterial stiffening.

## Methods

### Animals

The SM22-CreER^T2^(ki) transgenic mouse was kindly provided by Robert Feil. (Interfakultäres Institut für Biochemie, Universität Tübingen, Tübingen, Germany)^47^. *Itgav*^flox/flox^ mice were obtained from A. Lacy-Hulbert^1^ and maintained on a C57BL/6 background were crossed with SM22-CreER^T2^(ki) mice to generate SM22-CreER^T2^(ki)/*Itgav*^flox/flox^ double transgenic mice. Genotype were determined by PCR as described previously^1,47^. To generate α_v_^SMKO^ mice, *Itgav*^flox/flox^/SM22-CreER^T2^(ki)/+ mice were injected intraperitoneally with 1 mg of tamoxifen (Sigma #T5648) in 100 µL of peanut oil (Sigma #P2144) for 3 days. *Itgav*^flox/flox^ littermates injected for 3 days with tamoxifen were used as controls. All experiments were performed 4–6 weeks after tamoxifen injection at age 9–11 months in both male and female mice. The reduction in *Itgav* gene and α_v_ subunit expression in carotid arteries was 60% in α_v_^SMKO^ mice^4^. Alzet osmotic minipumps (Charles River, #AP-2004, L’Arbresle, France) containing Ang II (Sigma # A9525) were implanted in mice previously anaesthetized by isoflurane inhalation at 3.5% in 1 L/min oxygen and then maintained at 1.5% in 1 L/min oxygen during the intervention. Ang II was administered subcutaneously at 1.5 mg/kg/day for 28 days. Mice were euthanized via exsanguination under isoflurane anesthesia (1.5% in 1 L/min oxygen). All animal breeding and housing were conducted in accordance with French regulations and experimental guidelines of the European Community and the protocols were approved by the local Animal Ethics Committee of the University of Lorraine (N° 01638.01) and of Sorbonne University (N°10217), France. All animal work in this study followed ARRIVE guidelines.

### In vivo arterial mechanical parameters and composition

Measurement in vivo of arterial stiffness and histological procedures have been detailed previously. Briefly, blood pressure, carotid arterial diameter and its systolic-diastolic variations were measured with an echographic device. From the pressure-diameter curves and the media cross sectional area (MCSA), arterial distensibility (Dist), circumferential wall stress (WS) and incremental elastic modulus (Einc) were calculated. Dist and Einc were calculated at mean arterial pressure MAP (estimated pressure-dependent component). Curves were also compared by computing mean values in common range: 80–100 mmHg for AP and 800–1000 kPa for Einc (estimated pressure-independent component).

Carotid arteries were fixed under pressure and stained with Sirius red (Sigma #365548) for collagen and Weigert’s resorcin-fuchsin (Sigma #N-0638) for elastic fiber densities. Composition of the arterial wall and MCSA were measured by computerized image analysis.

### Cell culture

Aortic VSMCs isolated from the descending thoracic aorta of *Itgav*^flox/flox^ mice were grown in low-glucose (1 g/L) DMEM (Gibco #31885) supplemented with 10% fetal bovine serum (FBS) (Sigma #F7524). VSMCs were seeded at a density of 4 × 10^5^ cells/well into 6-well plates or 7.5 × 10^3^ cells/well into 8-well labtek and allowed to grow for 24 h. For α_v_ knock down (KD), VSMCs were incubated for 48 h with 0.5 µL/well of Cre-expressing adenovirus (10^10^ PFU/mL) in DMEM supplemented with 10% FBS (α_v_-KD cells), and the medium was replaced with 0.5% FBS supplemented DMEM. An adenovirus expressing the green fluorescent protein (GFP) was used as a control (empty vector – EV – cells). The expression of α_v_ integrin was reduced by 82% in α_v_-KD cells compared to EV cells^4^. VSMCs were then treated with saline or 200 nM Ang II for 48 h.

### Atomic force microscopy

Elasticity at cell level was investigated by atomic force microscopy (AFM)^12,13,15^. AFM measurements were performed on aortic VSMCs which grew on a glass substrate immersed in DMEM medium with 0.5% FBS to lead to dense monolayers of confluent cells. A multimode 9 AFM (Bruker, Palaiseau, France) was used in peak force mode to acquire AFM topographic cell images. The tips used were silicon Bruker Scanasyst-air tips (conical tips characterized by their angle: 10°), on a nitride cantilever of resonance frequency of 70 kHz. If the image quality was sufficient, indicating a correct attachment of the cell on the substrate, a virtual grid was superimposed on the image. Its pitch was between 0.5 μm and 1.5 μm. For each group of cells, between 71 and 116 measurements have been performed.

Force curves recorded according to the grid were taken at a speed of 1.5 Hz. They were corresponding to an overall vertical course of the sample below the tip of 300 nm but with no sample deformation under the tip larger than 50 nm (nanoindentation).

Thanks to the initial cantilever/tip calibration, the force curves were displayed as a function of the sample deformation. Force curves were fitted by a Sneddon model corresponding to the theoretical analysis of the indentation of a conical element into a sample^48^. Assessing 0.5 as value of Poisson coefficient, the fit allowed to extract the apparent elastic modulus of the cell.

### Computational model of VSMC stiffness

To simulate numerically an AFM indentation test on SMCs, we created a finite element (FE) model of an idealized SMC and of an indenting tip using the Abaqus^®^ explicit software. This model was introduced in a prior study^23^. The model has a semi-spindle shape of 250 × 30 µm^2^ including the cell membrane, cytoplasm, stress fibers, nucleus, and nuclear envelope. The cell membrane and nuclear envelope were modeled as thin layers, each with a thickness of 10 nm, covering the cytoplasm and nucleus, respectively. We also considered 20 stress fibers with a diameter of 0.2 μm spanning both extremities of the SMC, representing focal adhesion sites^49^. The cell lied on the surface of a substrate of dimension 0.4 × 5 × 5 mm^3^.

The cell membrane and nuclear envelope were modeled as elastic materials (near-incompressible NeoHookean elastic model taking into account finite strains) with a reference elastic modulus (3 times the shear modulus) of 3600 kPa, utilizing 3860 and 192 shell elements, respectively. The cytoplasm and nucleus were assumed to behave similarly but significantly softer with a reference elastic modulus (3 times the shear modulus) of 600 Pa^23^. They were discretized by 16,388 and 1024 brick elements, respectively. Lastly, stress fibers were modeled using linear elastic truss elements with elastic modulus of 50 MPa, capable of generating contractile forces. A 3.7 nN contractile force was assigned for each stress fiber according to our prior study^23^.

We assumed that SMC were tied on the surface of the substrate at focal adhesions, located on the cell membrane. The remaining part of the cell membrane had direct physical contact with its underlying substrate.

To simulate the AFM indentator, we defined analytical rigid elements modeling a conical tip with the half-angle of tip α = 20°, and we defined a surface-based contact between the tip and cell membrane in Abaqus.

FE simulations consisted of 2 steps. First, we applied gravitational load to the SMC and activated the basal tension of stress fibers^23^. Second, we simulated an indentation of 500 nm by vertically moving the tip down. We saved the computed contact force between the tip and the cell every 1 nm displacement of the tip in order to plot force-displacement curves.

From these simulated force-displacement curves, we derived the so called indentation apparent Young’s modulus, similarly to the one provided in actual AFM indentation. This indentation stiffness is derived by fitting the force-displacement curves with the Sneddon’s model, which may be written such as:

$$F = 0.31E{\delta ^2}$$

in which F and δ denote the measured force and penetration depth of the tip, respectively, and E represents the so called apparent Young’s modulus of the SMC under indentation testing. As it can be noted, the Sneddon model considers a quadratic relationship between force and displacement, factorized by E. Therefore, E was derived by linear regression of the Sneddon’s model onto the experimental or simulated force-displacement curves.

### Immunohistochemistry

Cells were fixed for 10 min with 4% paraformaldehyde solution at room temperature, washed three times for 5 min with PBS, and stained for 30 min with Alexa Fluor™ Plus 555 Phalloidin (Invitrogen #A30106) revealing filamentous actin (F-actin) and DAPI for nuclei staining. FAK and vinculin were revealed using rabbit primary antibodies (FAK (Thermofisher, #AHO0502 at 1:100 for FAK, and Invitrogen, #700062 at 1:1/100 for vinculin). Following three washes of 5 min each, cells were mounted and imaged with a Nikon Eclipse Ci fluorescent microscope (objective 40x). To assess whether there was a cortical accumulation in α_v_^SMKO^ VSMCs with Ang II, we computed a cortical fluorescence ratio i.e. the ratio of red fluorescence intensity (F-actin staining) near wall on mean intensity along lines crossing cells. In details, we defined lines crossing cells of fixed orientation (vertical, horizontal and 45° tilted) to obtain intensity profiles. A dedicated signal processing Matlab^®^ script was used to perform the following steps: filtering with an Hanning window (width 5 μm), cell limit detection, computation of the ratio max level near the wall (5 μm) on the mean level.

### Statistical analysis

All values were expressed as means ± SEM. Two-way ANOVA was used to compare the four experimental groups of mice. Differences were considered significant at values of *p* < 0.05.

## Data Availability

The data underlying this article will be shared on reasonable request to the corresponding author.

## Declarations

this paper to disclose.
